# P032 Giant cell arteritis masquerading as a hyperinflammatory syndrome

**DOI:** 10.1093/rap/rkag113.062

**Published:** 2026-11-03

**Authors:** Zehra Gul, Aqeel Anjum

**Affiliations:** Swansea Bay University Hospitals, Abertawe, United Kingdom; Swansea Bay University Hospitals, Abertawe, United Kingdom

## Abstract

**Introduction:**

Giant cell arteritis (GCA) is the commonest systemic vasculitis in older adults and classically presents with headache, scalp tenderness, jaw claudication and visual disturbance. However, atypical presentations dominated by constitutional symptoms and marked systemic inflammation may delay diagnosis. This challenge is amplified when alternative explanations for inflammation exist. We describe an 80-year-old man with ulcerative colitis recently treated with vedolizumab, who developed a severe hyperinflammatory syndrome with extensive negative investigations for infection and malignancy. The eventual diagnosis of ultrasound-confirmed GCA was only established following relapse after corticosteroid withdrawal.

**Case description:**

An 80-year-old man with ulcerative colitis, atrial fibrillation, diabetes mellitus, hypertension and hyperlipidaemia was admitted in March 2026 with profound fatigue, abdominal symptoms, fevers and a marked inflammatory response. Laboratory investigations demonstrated CRP 350 mg/L, white cell count 25 ×109/L, thrombocytosis exceeding 1000 ×109/L and ferritin 964 µg/L. He had recently commenced vedolizumab for inflammatory bowel disease (November 2025).

Extensive investigations for infection and malignancy were unrevealing. Blood cultures, urine cultures, viral screening, echocardiography and cross-sectional imaging failed to identify a cause. Gastrointestinal assessment did not demonstrate convincing evidence of active inflammatory bowel disease. In the absence of an alternative diagnosis, a severe hyperinflammatory syndrome was considered, with vedolizumab-related inflammation among the differential diagnoses.

He received prednisolone 40 mg daily with rapid clinical and biochemical improvement. Following corticosteroid withdrawal, however, he developed worsening fatigue, persistent diplopia, ankle swelling and recurrence of left-sided temporal headache. Review in rheumatology clinic revealed that headache symptoms had preceded steroid discontinuation and that diplopia had been present since the original admission.

Repeat investigations demonstrated recurrent inflammation with CRP 107 mg/L, platelet count 608 ×109/L and haemoglobin 120 g/L. Although he denied jaw claudication, scalp tenderness, tongue claudication or polymyalgia rheumatica symptoms, the combination of relapsing inflammation, headache and visual symptoms prompted urgent vascular ultrasound assessment.

Ultrasound demonstrated a halo sign within the left superficial temporal artery with increased intima-media thickness, confirming giant cell arteritis. High-dose prednisolone was recommenced, resulting in significant symptomatic improvement. The patient subsequently commenced a standard glucocorticoid taper with bone and gastric protection.

**Discussion:**

This case illustrates an atypical presentation of GCA in which profound systemic inflammation dominated the clinical picture and delayed recognition of vasculitis. The patient initially presented with fever, constitutional symptoms and markedly elevated inflammatory markers without the classical cranial manifestations typically associated with GCA. The degree of inflammation, including CRP 350 mg/L, thrombocytosis greater than 1000 ×109/L and ferritin approaching 1000 µg/L, prompted extensive investigation for infection, malignancy and inflammatory bowel disease activity.

Diagnostic complexity was further increased by the recent introduction of vedolizumab. The temporal association between biologic therapy and the onset of severe inflammation created anchoring bias towards a drug-related inflammatory syndrome. As a result, the possibility of vasculitis was not initially prioritised despite the presence of headache and diplopia, symptoms that were recognised retrospectively as important clues.

The subsequent relapse following corticosteroid withdrawal proved diagnostically valuable. Re-emergence of constitutional symptoms alongside persistent headache and visual disturbance prompted reassessment and expedited vascular ultrasound. Identification of a temporal artery halo sign provided rapid confirmation of GCA without the need for temporal artery biopsy and facilitated prompt treatment.

This case aligns with several themes highlighted by the BSR PMR/GCA/LVV case prompts, including delayed diagnosis, overlap with another inflammatory condition, diagnostic uncertainty created by biologic therapy, and the important role of imaging in establishing the diagnosis. It also demonstrates how GCA may present primarily as a pyrexia of unknown origin or hyperinflammatory syndrome.

Clinicians should maintain a high index of suspicion for GCA in older adults with unexplained inflammation, even in the absence of classic cranial symptoms and when alternative explanations appear plausible. Early consideration of vascular imaging may reduce diagnostic delay and prevent ischaemic complications.

**Key learning points:**

• This case highlights several important learning points relevant to contemporary GCA practice.

• First, GCA should remain an important differential diagnosis in older adults presenting with unexplained hyperinflammation, fever of unknown origin or marked thrombocytosis, even when classical cranial symptoms are absent. Constitutional symptoms may dominate the initial presentation and precede more recognisable manifestations of vasculitis.

• Second, coexistence of another inflammatory disease can obscure the diagnosis. In this patient, ulcerative colitis and recent vedolizumab exposure provided plausible alternative explanations for the inflammatory syndrome and contributed to diagnostic uncertainty. Awareness of potential cognitive biases, particularly diagnostic anchoring, is essential when evaluating complex patients.

• Third, visual symptoms warrant careful scrutiny even when they appear atypical. Persistent diplopia was present from the initial illness but was not initially attributed to GCA. Retrospective review demonstrated that key diagnostic clues were present earlier in the disease course.

• Fourth, vascular ultrasound played a pivotal role in establishing the diagnosis. Rapid access to imaging enabled confirmation of GCA through identification of a temporal artery halo sign and avoided further delay in treatment. This emphasises the value of modern fast-track pathways and ultrasound expertise in contemporary vasculitis services.

• Finally, relapse following corticosteroid withdrawal can provide important diagnostic information. The recurrence of symptoms and inflammatory markers following steroid cessation supported an underlying steroid-responsive inflammatory disorder and prompted re-evaluation of the original diagnosis.

• This case reinforces the need to consider GCA beyond its classical presentation and demonstrates how vascular imaging can facilitate diagnosis in diagnostically challenging cases.

